# Attention to Elderspeak: A Call for Dignity-Affirming Communication in Advanced Nursing Care

**DOI:** 10.3390/clinpract16010021

**Published:** 2026-01-22

**Authors:** Takahiko Nagamine

**Affiliations:** 1 Sunlight Brain Research Center, Hofu 747-0066, Japan; tnagamine@outlook.com; 2 Shoshido Geriatric Health Services Facility, Hofu 747-0066, Japan

**Keywords:** advanced nursing care, CHAT training, communication, elderspeak, Iowa Coding Scheme for Elderspeak (ICodE), resistiveness to care, patient safety

## Abstract

Elderspeak is a form of communication overaccommodation directed toward older adults, characterized by simplified language and an elevated pitch. While typically well-intentioned, it is rooted in ageist stereotypes and linked to negative health outcomes. A literature search was conducted in PubMed, CINAHL, and PsycINFO (2018–2025), yielding 24 key articles focusing on acute and surgical settings. The purpose of this narrative review is to synthesize current evidence on Elderspeak within acute care hospitals and propose a research framework and intervention strategies. Elderspeak is a key determinant of resistiveness to care (RTC), particularly in acute settings where it is triggered by functional impairment. Exposure increases patient distress and negatively impacts vital signs and cooperation with medical interventions. Inconsistent measurement is being addressed through standardized schemes like the Iowa Coding Scheme for Elderspeak (ICodE). This paper proposes that future research must employ mixed-methods, longitudinal designs to capture the impact of Elderspeak on long-term outcomes. Drawing on the ICodE, we propose a qualitative self-reflection tool for clinicians to enhance awareness in high-stakes acute settings. Eliminating Elderspeak is a foundational necessity for patient safety and dignity-affirming care in advanced nursing.

## 1. Introduction

Communication forms the core of therapeutic relationships, yet in the context of caring for older adults, a prevalent and often subtle pattern known as Elderspeak frequently undermines these essential interactions. Elderspeak is a form of communication overaccommodation directed toward older adults, rooted in implicit ageist stereotypes that often assume reduced cognitive or auditory capacities [1,2]. This phenomenon is not malicious; the intent behind using Elderspeak is typically benign—to convey warmth, comfort, or enhance clarity—but its consequence is demonstrably negative, as this style is widely perceived by older adults as patronizing, disrespectful, and infantilizing [3]. This pattern is a critical area of concern, particularly given the strong empirical links between Elderspeak and adverse outcomes for older adults, such as increased aggression and refusal of care [4,5]. The theoretical foundation for this concern is the Communication Predicament of Aging (CPA) model. This model posits that the mere presence of age-related cues triggers a stereotypical expectation of incompetence in the caregiver. This leads to speech modifications (Elderspeak) that constrain the older adult’s ability to respond effectively, eventually resulting in the loss of self-esteem and functional decline. While this cycle is well-studied in residential care, its manifestation in the fast-paced, high-stress acute hospital environment remains under-examined. Given that acute care requires rapid patient cooperation for successful medical and surgical outcomes, understanding how communication mediates this cooperation is a foundational necessity for modern nursing practice.

The pervasive nature of Elderspeak warrants increased scrutiny across all disciplines that interact with older adults. Studies have confirmed its presence not only in long-term care, but also, critically, in acute hospital settings where it is linked to patient functional impairment [4,6,7], and even in the educational environments of future healthcare professionals [8], suggesting a systemic, culturally ingrained problem. The paradox lies in the conflict between the caregiver’s positive intent and the negative, measurable impact on the recipient’s well-being. Therefore, the elimination of Elderspeak and the universal adoption of dignity-affirming communication is a necessary standard for all care of older adults. To effectively address this ageist behavior, research must move beyond mere identification to provide rigorous measurement, link communication to long-term outcomes, and develop tangible, effective alternatives.

This narrative review is structured to systematically define Elderspeak, summarize the current state of research—including established consequences, effective communicative alternatives, and persistent methodological challenges—and outline a comprehensive methodological approach for future research. It specifically emphasizes the implications of Elderspeak for advanced clinical nursing within the acute care setting, arguing that communication quality is an essential, modifiable component of patient safety and successful surgical and medical outcomes. It concludes by proposing a practical, qualitative self-reflection tool designed to enhance caregiver self-awareness and ultimately improve communication practices in high-stress clinical environments.

## 2. Materials and Methods

This narrative review synthesizes literature on Elderspeak by searching the PubMed, CINAHL, and PsycINFO databases. The search focused on peer-reviewed articles published between 2018 and 2025. The exact search strings included: (“Elderspeak” OR “Communication Predicament of Aging” OR “Communication Accommodation”) AND (“Acute Care” OR “Hospital” OR “Nursing” OR “Surgical Care”).

Inclusion criteria required papers to: (1) provide empirical data on Elderspeak in healthcare settings, (2) discuss theoretical frameworks of ageism, or (3) evaluate communication interventions. A total of 24 articles were reviewed. While this number is specific, it reflects the paucity of research specifically linking Elderspeak to acute surgical outcomes compared to the more extensive literature in long-term care. This selection allows for a targeted analysis of the “bridge” between communication and acute nursing care. To ensure the rigor and reproducibility of this synthesis, the literature selection process followed a standardized screening protocol. After the initial database search, titles and abstracts were screened for relevance by the author, followed by a full-text review to ensure each included study provided specific linguistic or behavioral data pertinent to Elderspeak. Data extraction focused on three primary domains: the linguistic triggers of Elderspeak, the measurable physiological/behavioral responses of patients (e.g., RTC), and the settings of the interaction (long-term vs. acute care). This systematic approach allowed for the identification of core themes despite the heterogeneous nature of the studies reviewed.

## 3. The Current State of Elderspeak Research: Focus on Acute Care Implications

Research has consistently established that Elderspeak is a pervasive, ageist behavior with clear negative consequences, significantly influencing the quality of care and the well-being of older adults. The consequences of Elderspeak pose a direct threat to the goals of advanced medical and surgical nursing in acute settings.

### 3.1. Definition, Linguistic Characteristics, and Theoretical Basis

Elderspeak is fundamentally an issue of communication overaccommodation, where a speaker modifies their speech based on perceived, often inaccurate, stereotypes of the listener’s age-related capacities [9]. This modification is manifested through specific paralinguistic and linguistic features, which collectively convey a message of dependency and incompetence. While Elderspeak is broadly defined as overaccommodation, inconsistencies arise in how researchers categorize its components. For example, some models focus strictly on prosodic features (pitch/intonation), while others emphasize lexical choices (terms of endearment). What is perceived as “warmth” by one practitioner may be coded as “infantilization” by another, creating a challenge for standardized measurement across different clinical cultures. The key characteristics of Elderspeak are detailed in Table 1.

In such studies, Elderspeak is measured with the detrimental effects of elderspeak being explained through the communication predicament of aging model [9]. This model describes a negative feedback loop: a younger speaker, observing cues of old age or, critically, signs of acute functional impairment (a common feature in the hospital setting), activates ageist stereotypes, leading to the use of Elderspeak (overaccommodation). The older adult, feeling demeaned, responds with withdrawal, behavioral resistance (RTC), or an adoption of dependent behaviors. This negative response then inadvertently reinforces the speaker’s original ageist assumptions, perpetuating the cycle and contributing to a broader spiral of decline in physical, cognitive, and functional status [5]. Elderspeak is thus conceptualized as a mechanism of systemic ageism within care environments, which can be acutely damaging in the time-sensitive and high-stakes environment of advanced clinical nursing.

### 3.2. Empirical Consequences and Impact on Acute Care Outcomes

A substantial body of research has quantified the adverse impact of Elderspeak, demonstrating a clear link between this communication style and negative health and behavioral outcomes, with direct implications for acute and surgical recovery. The most critical and consistently cited finding is the direct link between Elderspeak and an increase in resistiveness to care (RTC), particularly in individuals living with dementia [3,6]. Observational studies have shown that the use of Elderspeak can effectively double the odds of a resident exhibiting RTC, which includes physical resistance, emotional distress, or refusal to cooperate with essential care tasks [4]. This strong evidence positions Elderspeak as a major, yet modifiable, trigger for challenging behaviors. In an acute setting, RTC can be catastrophic, leading to patient refusal of vital monitoring, medication administration, ambulation, or post-surgical wound care—all of which are critical to preventing complications and enhancing patient outcomes. This necessitates that advanced nursing practice view the elimination of Elderspeak as a patient safety intervention.

The prevalence of Elderspeak is a growing concern outside of long-term care. Studies have demonstrated its occurrence in hospital settings [2,6], and, critically, in acute general and geriatric hospitals [7]. The research by Schnabel et al. [7] found common Elderspeak features like diminutives and tag questions in acute settings, suggesting that functional impairment (e.g., following a stroke, surgery, or during a severe illness) may be a more salient trigger for staff’s ageist stereotypes than cognitive impairment alone. This highlights that Elderspeak is not merely a long-term care issue but a systemic problem across the continuum of care, requiring targeted intervention in acute nursing education and protocols. Worryingly, Elderspeak is also found among students in healthcare education programs. For instance, in a study of simulated provider-patient encounters with preclinical chiropractic students, Elderspeak was evident, with the use of collective pronouns (e.g., “we”) being the most common form [8]. This confirms the need for mandatory anti-ageism training, focusing on communication, throughout professional education to prevent the transmission of ageist behaviors into clinical practice.

Furthermore, chronic exposure to Elderspeak has profound pathophysiological consequences. The feeling of being patronized or disrespected by a caregiver—an authoritative figure—is a form of interpersonal stress. This stress can trigger the sympathetic nervous system, activating the hypothalamic–pituitary–adrenal axis and leading to an increased release of cortisol and catecholamines. In an acute care environment, this constant state of vigilance and agitation can negatively impact crucial health parameters, including increasing heart rate, blood pressure, and respiratory rate, which may complicate the management of complex medical conditions or post-surgical recovery. Moreover, the psychological impact of chronic exposure to Elderspeak leads to negative self-perceptions, decreased self-esteem, social withdrawal, and increased symptoms of agitation, aggression, depression, and anxiety [10,11]. This agitation, fueled by disrespectful communication, can directly increase the patient’s subjective experience of pain and necessitate higher doses of analgesia, thereby compromising complex pain management protocols.

It is important to note, however, that the perception of certain Elderspeak features is not entirely uniform. For instance, a qualitative study exploring the use of terms of endearment in assisted living found that older adults’ perceptions were highly individualized, with some viewing the terms positively or neutrally, while others found them childish or disrespectful [12]. This finding suggests that while the overall pattern of Elderspeak is harmful, person-centered care must still account for individual preferences, underscoring the complexity of communication interventions and the necessity of thorough clinical assessment prior to establishing a communication baseline. A key result of this synthesis is the identification of a significant research gap: while Elderspeak is extensively documented in chronic care, its impact in the acute phase is currently supported primarily by case-level evidence and emerging observational studies. Analysis of these acute-phase cases reveals a critical pattern: even in high-acuity environments, Elderspeak acts as a primary trigger for ‘Resistiveness to Care’ (RTC). For instance, specific case observations indicate that during time-sensitive procedures (e.g., post-surgical wound care), the use of infantilizing language leads to an immediate increase in patient agitation and a subsequent refusal of vital interventions. This suggests that the behavioral impact of Elderspeak is not attenuated by the clinical urgency of the acute setting; rather, it may be exacerbated by the patient’s heightened vulnerability.

### 3.3. Alternatives, Language Stimulation, and Evidence-Based Interventions

In such a study, Elderspeak will be measured using direct audio/video observation While much research focuses on the problem, emerging work offers concrete alternatives and solutions essential for advanced nursing practice. A qualitative study analyzing successful communication between older Catholic nuns and their caregivers identified three genres of interaction that avoided Elderspeak while maintaining lexically and grammatically rich communication [13]. These successful alternatives included: (a) offered and requested blessings, (b) jokes, and (c) narratives [13]. These strategies offer practical models for caregivers to engage older adults, particularly those with cognitive or physical challenges, in complex interactions that affirm dignity and may reduce resistiveness to care. In fact, the direct stimulation of language may be a viable intervention path to combat the simplification inherent in Elderspeak. Preliminary case series studies have investigated the effectiveness of language stimulation programs (LSP) for institutionalized older people. While small, these studies suggest that speech-language therapy interventions through an LSP can contribute to improving the cognitive-linguistic performance of institutionalized elderly, potentially mitigating the long-term effects of linguistic deprivation or oversimplified communication [14]. Such findings reinforce the need for communication strategies that elevate, rather than diminish, the complexity of interaction with older adults.

The most promising development is the validation and implementation of standardized tools and training. First, the rigorous validation of systematic tools, such as the Iowa Coding Scheme for Elderspeak (ICodE) [15], now provides an evidence-based standard to reliably and validly document the use of Elderspeak. Further validation established its excellent inter-rater and intra-rater reliability, demonstrating that ten of the eleven Elderspeak attributes coded by ICodE were perceived by older adults as more patronizing or less respectful, and eight were linked to increased rejection of care by individuals with dementia [16]. This tool is crucial for research and for auditing communication quality in advanced clinical settings.

Second, communication-focused interventions like CHAT training (Communication, Hearing, and Awareness Training) have been shown to be effective in various settings, leading to a measurable reduction in staff Elderspeak and an associated reduction in resident resistiveness to care [17,18,19]. The successful adaptation of these programs across different long-term services and support settings confirms the feasibility of scalable education [17]. Implementing such structured, evidence-based training across the interdisciplinary team—including advanced practice nurses, physicians, therapists, and surgical support staff—is a clear directive for improving communication quality in acute care.

Third, the use of simulation as a valid methodology for both research and training is gaining traction. A recent mixed-methods pilot study found that Elderspeak communication elicited in high-fidelity dementia care simulations was congruent to communication produced in actual patient care, demonstrating that simulation is a valid and feasible method for both research and person-centered communication training for nursing staff [20]. This is a critical development for educational programs, including those where Elderspeak has been noted, such as in preclinical chiropractic training [8]. High-fidelity simulation offers a safe environment for advanced nurses to practice dignity-affirming communication skills and receive debriefing, thereby fostering expertise in a foundational element of quality care.

## 4. The Path Forward: Integrating Communication Quality into Advanced Clinical Nursing

Advancing the field requires a comprehensive, systematic approach that moves beyond documenting immediate negative reactions to fully investigate the sustained impact of this communication style on a resident’s life and recovery trajectory. The integration of advanced surgical and medical nursing requires a holistic view of the patient, in which communication is recognized as a key therapeutic modality.

### 4.1. Rationale for Longitudinal Study and Comprehensive Outcomes

A comprehensive, long-term approach is required to fully capture the sustained impact of Elderspeak. Elderspeak’s link to RTC directly impedes essential Activities of Daily Living (ADL) care, making functional status (ADLs/IADLs) a sensitive health indicator. Chronic exposure and the associated stress-response in the acute setting may lead to non-cooperation and a prolonged functional decline, delaying discharge and increasing the risk of re-hospitalization. Furthermore, Quality of Life (QoL) is a critical, multidimensional outcome, separate from quality of care [21]. Elderspeak, by being patronizing and reinforcing power differentials, directly threatens core QoL domains such as autonomy, dignity, individuality, and meaningful social engagement. Therefore, future research must employ a methodology capable of linking communication patterns to long-term changes in these critical areas. Specifically, a shift from cross-sectional or short-term intervention studies to longitudinal designs is imperative to facilitate the translation of evidence into practice between communication patterns and chronic health consequences. Moreover, interventions should be designed not just to reduce the negative (Elderspeak), but to promote the positive (rich, complex communication), potentially by integrating techniques identified as successful alternatives (blessings, narratives, jokes) [13] or by incorporating formal language stimulation components [14].

### 4.2. Framework for Future Investigations

To advance the field, future studies should adopt mixed-methods, longitudinal designs [22]. Such a design would be the most robust approach to investigate the impact of Elderspeak on health status. Future research should measure Elderspeak using direct audio/video observation coded with validated tools like the ICodE [15,16]. Researchers should implement sequential analyses to link specific instances of Elderspeak to immediate behavioral responses.

### 4.3. Enhancing Caregiver Self-Awareness for Advanced Nursing Practice

Because Elderspeak is a widespread communication pattern often used unconsciously by caregivers, a key intervention is to increase self-awareness [10]. Caregivers, especially those in the high-stress, fast-paced acute care environment, often struggle to recognize their own use of Elderspeak. Therefore, self-assessment is an essential prerequisite for effective behavioral change. The goal is to objectively and systematically observe one’s own communication patterns, without self-blame [22]. While existing communication measurement tools exist for institutional settings, they often focus on quantitative frequency [23]. The tool proposed in Table 2 is unique because it is designed for qualitative self-reflection in the high-stress acute care environment [24]. This tool was systematized by aligning the validated attributes of the ICodE with common clinical scenarios observed in hospital settings, providing a bridge between coding schemes and real-time bedside behavior.

This table, which creates self-questions from the elements, represents a qualitative approach and has the potential to elicit rich, descriptive responses regarding communication practices and underlying beliefs. The self-questions promote a deeper level of engagement and reflection that cannot be achieved through simple numerical scores. This approach is consistent with the needs identified in successful training programs that emphasize awareness [17,18]. Such questions may be used to raise awareness of Elderspeak through group discussions and reflection sessions, for example, during post-simulation debriefing protocols [20]. Facilitators can use structured prompts to introduce caregivers to the concept of Elderspeak and encourage them to use their newfound awareness as a tool to improve communication, reduce resistance to caregiving, and intentionally explore enriching and dignity-affirming communication alternatives [13]. Integrating such self-reflection into daily practice is a necessary component of advanced clinical nursing competency, ensuring that communication becomes a therapeutic intervention rather than a barrier to high-quality care. While the proposed self-reflection tool (Table 2) and the recommended research framework offer a path forward for advanced nursing, several limitations must be acknowledged. First, the self-reflection questionnaire is currently a qualitative, formative tool rather than a psychometrically validated scale. Its primary purpose is to trigger clinical mindfulness rather than to provide a standardized score. Future research should focus on validating this tool against objective coding (such as the ICodE) to determine its accuracy in changing caregiver behavior.

## 5. Conclusions and Future Directions

Elderspeak is a profound issue in gerontological care, representing a pervasive, yet modifiable, ageist behavior that inflicts measurable harm on older adults by driving behavioral resistance (RTC), eroding self-esteem, and compromising the quality of care. The universal commitment to eliminating Elderspeak is a necessary standard for all care of older adults, especially within the high-stakes environment of acute and advanced clinical nursing, where non-cooperation can directly compromise patient safety and recovery from complex surgical or medical procedures.

The field has matured, moving from problem identification to the development and validation of rigorous methods for its measurement and control (e.g., the ICodE [16]), and scalable intervention strategies (CHAT training [18]). Furthermore, research is starting to identify concrete, rich, and dignity-affirming communication alternatives to Elderspeak [13], opening a path for more holistic interventions that focus on both reduction of the negative and promotion of the positive. The widespread prevalence of this behavior across diverse settings, from long-term care to acute hospitals [7] and professional education [8], underscores the need for a systemic response anchored in evidence-based practice.

Moving forward, the research agenda must focus on three interconnected areas to support advances in clinical nursing. First, continued refinement and adoption of standardized coding tools are essential to maintain rigor and comparability across studies, allowing nursing leadership to audit and improve communication quality. Second, there is a critical need to implement longitudinal, mixed-methods research to systematically link communication exposure to sustained changes in health status, functional recovery, and quality of life. This research must explore not only the impact of reducing Elderspeak but also the potential for interventions like language stimulation programs to mitigate cognitive decline [14]. Finally, the widespread utilization of practical, qualitative self-awareness tools, such as the proposed Self-Reflection Questionnaire, is necessary to empower formal and informal caregivers to recognize their unconscious biases and adopt person-centered communication styles. By integrating rigorous, standardized research with effective, reflective intervention tools, the healthcare community can successfully challenge ageist communication patterns and move toward a truly person-centered approach that affirms the dignity and autonomy of every older adult, thereby optimizing outcomes in the advanced clinical setting.

## Figures and Tables

**Table 1:** Key Linguistic Characteristics and Examples of Elderspeak.

Characteristic	Definition	Examples
Juvenile Lexical Choices	Use of words or phrases typically reserved for children, or terms of endearment that infantilize.	“Sweetie,” “Honey,” “Buddy,” “Good girl/boy,” “Now, now”
Exaggerated Prosody	Overly high-pitched voice, exaggerated intonation, or overnurturing tone.	“Baby talk” sound, overly singsong voice.
Collective Pronoun Substitution	Using “we” instead of “you” when addressing the resident, implying shared action or dependency.	“Are we ready for our bath now?”, “Let’s eat our dinner.”
Simplified Syntax/Sentence Length	Use of overly short, simple sentences, often below the resident’s cognitive capacity.	“Eat this,” “Walk now.”
Minimizing Words/Mitigating Expressions	Words that downplay the resident’s concerns or feelings.	“It’s okay,” “Don’t worry,” “Just a little poke.”
Exaggerated Praise	Comments praising patients that are overdone, repeated, or seem inauthentic.	“That’s a very good job, sweetie!” for a simple task.
Tag Questions	A question is asked while providing the desired answer, restricting choice despite the appearance of offering it.	“You’re ready for breakfast now, aren’t you.
Belittling Actions/Comments	Staff laughter at the patient (not with), ignoring, interrupting, or demeaning comments.	“You’re pretty forgetful now, aren’t you [laughs].”

Note: This table provides a clear, concise reference for the specific attributes of Elderspeak that will be measured, aiding in both data collection and the interpretation of findings. It operationalizes the primary independent variable for the study by consolidating diverse descriptions of Elderspeak into a structured format, thereby guiding researchers in consistently identifying and coding Elderspeak during the analysis of recorded interactions.

**Table 2:** Proposed Qualitative Self-Reflection Tool for Clinicians.

Elements	Self-Questioning
Terms of Endearment/Nicknames	Do you ever use terms like “honey,” “dear,” “sweetie,” “grandma,” or “gramps” when addressing older adults who are not close family or friends? If so, in what contexts, and what is your intention behind using them?
Simplified Language/Vocabulary	Do you sometimes simplify your vocabulary or sentence structure when speaking to older adults?
Speech Rate and Volume	Do you consciously adjust your speaking rate (slower) or volume (louder) when talking to older adults?
Pitch and Intonation	Have you noticed yourself using a higher pitch or a “singsong” (overly nurturing) tone when speaking with older adults?
Collective Pronouns (“We”)	Do you ever use phrases like “Are we ready for our bath?” or “It’s time for us to take our medicine now” instead of “Are you ready...” or “It’s time for you...”? If so, in what situations and why?
Tag Questions/Checking for Understanding	Do you frequently use tag questions (e.g., “You’re ready, aren’t you?”) or overtly check for understanding (e.g., “Do you understand what I’m saying?”) with older adults?
Repetition and Paraphrasing	When do you find yourself repeating or rephrasing information for older adults?
Focus on Tasks vs. Conversation	Do you find yourself focusing more on giving instructions or completing tasks with older adults, sometimes at the expense of engaging in reciprocal conversation?

Note: This tool is designed for formative self-assessment and educational debriefing. It has not yet undergone psychometric validation for quantitative measurement.

## Data Availability

No new data were created or analyzed in this study.

## Abbreviations

The following abbreviations are used in this manuscript:

ADL	Activities of Daily Living
CHAT	Communication, Hearing, and Awareness
ICodE	Iowa Coding Scheme for Elderspeak
LSP	language stimulation programs
QoL	Quality of Life
RTC	resistiveness to care
